# Exploring the predictability of distributed lag nonlinear models using SARS-CoV-2 wastewater-based surveillance in multiple communities in Alberta, Canada

**DOI:** 10.1371/journal.pone.0349030

**Published:** 2026-07-10

**Authors:** Rhonda J. Rosychuk, Bonita E. Lee, Judy Y. Qiu, Tiejun Gao, Michael D. Parkins, Casey R. J. Hubert, Xiaoli Pang

**Affiliations:** 1 Department of Pediatrics, Faculty of Medicine and Dentistry, College of Health Sciences, University of Alberta, Edmonton, Alberta, Canada; 2 Department of Mathematical and Statistical Sciences, Faculty of Science, College of Natural and Applied Sciences, University of Alberta, Edmonton, Alberta, Canada; 3 Department of Statistics and Actuarial Science, Faculty of Science, Simon Fraser University, Burnaby, British Columbia, Canada; 4 Women and Children’s Health Research Institute, Edmonton, Alberta, Canada; 5 Alberta Precision Laboratory, Public Health Laboratory, Edmonton, Alberta, Canada; 6 Department of Laboratory Medicine and Pathology, University of Alberta, Edmonton, Alberta, Canada; 7 Li Ka Shing Institute of Virology, University of Alberta, Edmonton, Alberta, Canada; 8 Department of Microbiology, Immunology and Infectious Diseases, University of Calgary, Calgary, Alberta, Canada; 9 Department of Biological Sciences, University of Calgary, Calgary, Alberta, Canada; 10 Advancing Canadian Wastewater Assets, University of Calgary, Calgary, Alberta, Canada; National Research and Innovation Agency, INDONESIA

## Abstract

**Background:**

Wastewater–based surveillance can be an important part of pandemic management, especially when testing capacity of individuals is limited. Statistical modeling can be used to examine the relationship between wastewater pathogen levels and clinical cases. The objective of this study was to examine the utility of distributed lag nonlinear modeling to derive the relationship between wastewater SARS-CoV-2 RNA levels and COVID-19 clinical cases across communities in Alberta, Canada when clinical testing was comprehensive.

**Methods:**

This retrospective cohort study used data from 24-hour composite wastewater collected and tested two to three times per week from 11 wastewater treatment plants (WWTPs) in Alberta, Canada during May 10, 2020, to March 15, 2022. The number of daily new cases of COVID-19 downloaded from Alberta Health’s centralized dataset of clinical surveillance of COVID-19 were mapped to each sewershed. Distributed lag nonlinear models were fit to describe the exposure-response relationship between the 7-day rolling average of SARS-CoV-2 RNA and daily new cases for each WWTP separately.

**Results:**

The 11 WWTPs served a population of 3,422,062 (77% of Alberta’s population) and 386,528 cases were documented during the study period. From 2021 onward, peaks in both wastewater viral RNA levels and cases tracked reasonably well. For almost all WWTPs, the best fitting model was a Poisson additive model with a P-spline for time. Models for the larger communities had better fits than smaller communities as represented by adjusted pseudo-R^2^ ranging from 80.7% to 94.4%. Models followed the same general trends as the actual COVID-19 cases over time.

**Conclusions:**

With relationships between wastewater viral RNA levels for SARS-CoV-2 and COVID-19 cases expected to vary over time and to be non-linear, distributed lag nonlinear models are promising. While the form of the models was similar across WWTPs, the resulting estimates were different among sites suggesting site-specific analyses are essential.

## Introduction

Surveillance for infectious agents in environmental water matrices and wastewater (sewage) was implemented as part of World Health Organization global eradication of polio program in the early 2000s [1,2]. With the advancement of molecular technology, quantification and characterization of pathogens in wastewater beyond qualitative detection became possible [3–5]. The discovery that people infected with severe acute respiratory syndrome coronavirus (SARS-CoV-2) shed the virus in their faeces led to advances in detecting and quantifying the virus in community wastewater [6]. Wastewater-based surveillance (WBS) for SARS-CoV-2 became part of pandemic management, which is especially useful when testing capacity was limited, and is an important tool to monitor disease burden in a jurisdiction and provide actionable information for public health officials [7–9]. Crucial to the usefulness of WBS is appropriate statistical approaches to quantify the relationships between wastewater viral nucleic acids levels and documented cases of disease.

A variety of approaches have been used to characterize the relationships between wastewater viral RNA levels and clinical COVID-19 cases including: correlation coefficients (Pearson and Spearman) with or without considerations of lags between the cases and wastewater measurements; regression techniques such as simple univariate and multivariate linear regression, autoregressive integrated moving average (ARIMA), and vector autoregression models; susceptible-exposed-infectious-recovered (SEIR) models; artificial neural networks; and generalized additive models [10]. Because the relationships between wastewater viral RNA levels and COVID-19 cases are likely time-varying, distributed lag models (DLMs) have been used [11,12]. When the relationship was nonlinear, distributed lag nonlinear models (DLNMs) have been used to model SARS-CoV-2 wastewater viral signals and COVID-19 hospitalizations in a single jurisdiction [13].

The objective of this paper is to use DLNMs to model and explore the association of COVID-19 cases and wastewater viral RNA levels for SARS-CoV-2 across all major urban centres within a jurisdiction. SARS-CoV-2 WBS data and COVID-19 clinical case counts in Alberta are unique datasets to be used to examine the application of DLNMs as Alberta performed the highest number of COVID-19 tests per population in Canada until the emergence of Omicron [14]. We determine separate DLNMs for each of 11 wastewater treatment plants (WWTPs) and a virtual WWTP – created to represent the provincial picture for Alberta, Canada – and compare and contrast the resulting models as the COVID-19 pandemic evolved over space and time.

## Materials and methods

### Study design

This retrospective cohort study used data from 11 WWTPs and the Alberta, Canada communities they serve during May 10, 2020, to March 15, 2022. These WWTPs were previously enrolled to be representative across different municipality characteristics (e.g., geographic location, population size, urban/rural) [9] and covered 77% of the population of Alberta [15]. A separate virtual WWTP was created by using the data combined from nine of the 11 sites, as two sites stopped data collection in 2021. Thus, a total of 12 WWTPs were included in our analyses [16].

### Ethics

This study was approved by the University of Alberta Research Ethics Board (Pro001847). The requirement for informed consent was waived. No patient was contacted.

### Collection and testing of wastewater samples

Operators of each WWTP collected a 24-hour composite sample two to three times per week. Before January 19, 2021, the samples were frozen and after that date, samples were stored at 4°C to improve the stability of SARS-CoV-2 ribonucleic acid (RNA) in the wastewater samples after further optimization and validation.[6]. Weekly shipping occurred until the first week of October 2021. After that time, samples were shipped three times a week to support real-time testing and reporting of results.

Processing and testing of wastewater samples were performed as described previously [6–8]. The results were expressed as genome equivalent copy numbers per 100 ml of wastewater. The average of the SARS-CoV-2 RNA level as detected by N1 and N2 gene was used in the analyses. These data were shared in real time with the public via the COVID Data Tracker website (https://covid-tracker.chi-csm.ca/).

### Population served by WWTP and COVID-19 cases

Alberta is divided into small, Local Geographic Areas (LGA) by Alberta Health and Alberta Health Services who have calculated the population for each LGA. The operators of each plant provided the geographic locations of the communities served. These service areas were mapped to the LGAs to determine the populations served by each WWTP. The number of daily new cases of COVID-19 for each LGA since March 2020 was downloaded from Alberta Health’s centralized dataset of clinical surveillance of COVID-19 by the date of report for these cases and compiled to represent new COVID-19 cases for the specific population served by each WWTP. The cases data were access for research purposes on June 29, 2022.

### Analysis

Only wastewater samples with positive SARS-CoV-2 results and quantified level of SARS-CoV-2 RNA in wastewater samples for each of the WWTP were included in the analyses. To reduce the noise of signal due to inherent variations with wastewater testing, 7-day rolling average of SARS-CoV-2 RNA level (genome copies/100 ml) were generated for each WWTP and population of the sewershed. This level is referred to as the wastewater viral RNA level in the rest of the paper.

Analyses were conducted separately for each WWTP because each community has a different pattern of water use. Distributed lag nonlinear models (DLNMs) [17] were fit to describe the exposure-response relationship. These models assume the relationship between wastewater viral RNA levels and clinical cases to be specified as nonlinear and delayed as captured through cross-basis functions. Furthermore, these models assume a maximum lag and require outcome data to arise from exponential family distributions (e.g., Poisson). The approach used cross-basis functions that are used in regression models as the predictor variables instead of the original variables. As cross-basis functions, we considered polynomial functions for the wastewater viral RNA level of up to degree 5 and similarly considered polynomial functions of up to degree 5 in the lag dimension. We assumed the lag ranged from 0 to 7 days based on reports in the literature: Delta and Omicron variants having lags of 5–14 and 1–4 days, [18] respectively, and 0–15 days being used for wastewater signals to hospitalization [13]. The cross-basis functions were used in a Poisson additive model with a time-varying intercept constructed as a penalized spline of time (days) [19,20]. While the formal mathematical formulation for general DLNMs is provided elsewhere [17], our specific Poisson additive model is similar to [13] and can be written as


$$\begin{array}{c} \text{log}(E\left(Y_{t}|W_{t}\right))=\alpha +f(t)+S(W_{t};\beta ) \end{array}$$

for clinical cases$Y_{t}$ over day t=1,….,n where α is a fixed intercept, f(t) is the penalized spline of time, $W_{t}$ is the wastewater viral RNA levels on day t,t−1,…,t−ℓ (where ℓ is the lag; up to 7 days as above), and $S\left(W_{t};\beta \right)$ is the wastewater viral RNA levels transformed into the cross-basis function with parameters β. The $S\left(W_{t};\beta \right)$ was specified as polynomial functions (up to degree 5 for wastewater viral level dimension, up to degree 5 for lag dimension). In addition, Poisson regression models were also fit with f(t)changed to a natural spline of time.

We fit 200 Poisson additive models and 200 Poisson regression models for each WWTP as we cycled through 1–5 degree polynomials for the wastewater viral RNA dimension, 1–5 degree polynomials for the lag dimension, and maximum lags of 0–7 days. Within the models for a WWTP, the model with the lowest Akaike information criterion (AIC) [21] was chosen as the final model for each WWTP. If the final model was a Poisson additive model, the pseudo-R^2^ was calculated. Relative risk (RR) estimates are obtained from the models at different lags and wastewater levels with the reference being at approximately the mid-value of the wastewater viral RNA level. This reference was WWTP-specific as the different populations served resulted in different ranges of wastewater viral RNA levels. A RR > 1 indicates that an increase in cases was associated with an increase in wastewater viral RNA levels and an RR < 1 indicates that a decrease in cases was associated with an increase in wastewater burden, relative to the reference. The predicted values from the models were plotted against the original data. All analyses were performed in R [22] with the dlnm [23] and mgcv [24] packages.

## Results

**Fig 1:**
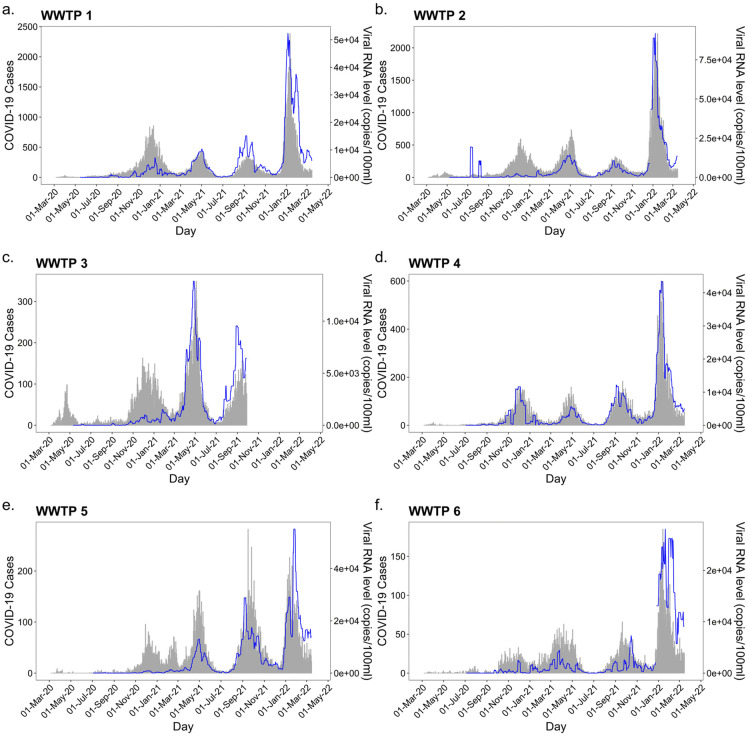
7-day rolling average wastewater viral RNA level (genome copies/100 ml) in wastewater (blue line) and clinical cases (grey) for WWTPs 1–6.

**Fig 2:**
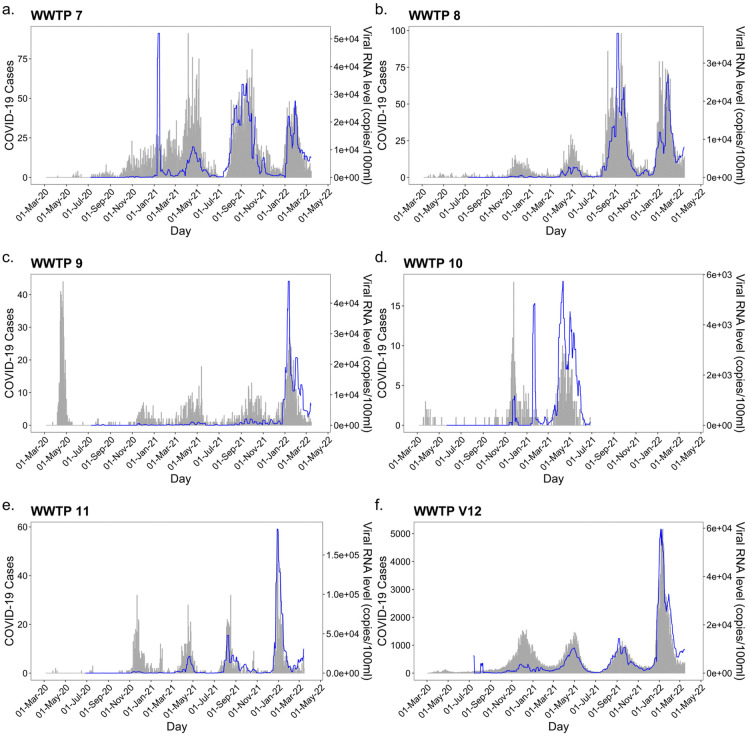
7-day rolling average wastewater viral RNA level (genome copies/100 ml) in wastewater (blue line) and clinical cases (grey) for WWTPs 7 to V12.

Eleven WWTPs from across the province and one virtual WWTP (created by using the data combined from nine of the sites) were included in the study and their characteristics summarized in Table 1. Not all WWTPs collected samples during the entire study period. Altogether, the population served was 3,422,062 and the total number of new COVID-19 cases reported during the study period was 386,528. The wastewater SARS-CoV-2 RNA levels varied overtime for each WWTP and the median number of new COVID-19 cases per day ranged from 0 to 98 across the 11 sewersheds and 323 for the virtual WWTP. Fig 1 and 2 show the median wastewater RNA viral level and the median and total number of COVID-19 cases for each WWTP over the study period. During the last half of the study (i.e., 2021 onward) the peaks in both wastewater viral level and COVID-19 cases appear to track reasonably well for the vast majority of sites.

**Table 1:** Characteristics of the wastewater treatment plants (WWTP), population served, and COVID-19 cases during the wastewater collection periods. V12 is a collection of the 9 starred WWTPs with data collection ending on March 15, 2022.

WWTP code	WWTP name	City/Town/County served	Population served	Start-End date (number of months)	Median SARS-CoV-2 RNA level in gc/mL (IQR)	Median new COVID-19 case per day (IQR)	Total number of new COVID-19 cases reported
1*	EPCOR Gold Bar	Edmonton, Leduc, Beaumont	1,115,021	18-May-20–15-Mar-22 (n = 23)	1697 (475-5320)	96 (44-254)	134,034
2*	The City of Calgary 01	Calgary North, Cochrane, Airdrie, Chestermere	1,104,208	10-May-20–15-Mar-22 (n = 23)	1715 (325-6288)	98 (40-239)	138,463
3	The City of Calgary 02	Calgary South	398,544	10-May-20–28-Sep-21 (n = 17)	518 (18-1781)	30 (8-77)	26,010
4*	Alberta Capital Region Wastewater Commission	Fort Saskatchewan, St. Albert, Spruce Grove, Strathcona County (including Sherwood Park), Sturgeon County, Stony Plain, Morinville, Bon Accord; Gibbons	326,497	6-Jul-20–15-Mar-22 (n = 20)	1570 (390-5170)	30 (8-75)	34,438
5*	The City of Red Deer	Red Deer, Sylvan Lake, Olds, Lacombe, Innisfail	187,857	6-Jul-20–15-Mar-22 (n = 20)	1208 (147-7590)	22 (5-49)	22,559
6*	The City of Lethbridge	Lethbridge	100,655	8-Jul-20–15-Mar-22 (n = 20)	617 (95-1795)	13 (4-25)	11,774
7*	Aquatera	Grande Prairie	74,245	10-Jul-20–15-Mar-22 (n = 20)	1187 (170-7475)	8 (3-20)	8,623
8*	The City of Medicine Hat	Medicine Hat	68,115	13-Jul-20–15-Mar-22 (n = 20)	399 (24-2918)	4 (1-13)	7,025
9*	High River Treatment Facility	Town of High River	16,922	12-Jul-20–15-Mar-22 (n = 20)	164 (0-798)	1 (0-3)	1,451
10	EPCOR Canmore	Town of Canmore	16,547	25-May-20–28-Jun-21 (n = 14)	0 (0-334)	0 (0-1)	425
11*	The Town of Banff	Town of Banff	13,451	30-Jun-20–15-Mar-22 (n = 20)	751 (48-6392)	0 (0-3)	1,726
V12 (Nine sites with *)	NA	All of the above except Calgary South & Town of Canmore	3,006,971	13-Jul-20–15-Mar-22 (n = 20)	2189 (846-6636)	323 (160-780)	358,195

After analyses of 200 Poisson additive and 200 Poisson regression models for each WWTP, the final models chosen based on the lowest AIC were summarized for WWTPs 1, 5, and 10 in S1, S2, and S3 Tables, respectively. Characteristics of the final models for each WWTP appear in Table 2. Almost all WWTPs had the same type of model form. For 10/12 WWTPs, the best fitting model was a Poisson additive model with a P-spline for time. For two WWTP s (WWTP 10 Canmore, WWTP 11 Banff), the best model was a Poisson regression model with a natural spline for time. Models for the larger served communities had better fits than the smaller communities as represented by adjusted pseudo-R^2^ ranging from 80.7% to 95.1%.

**Table 2:** Characteristics of the final DLNMs for each WWTP: model type, degree of polynomial for wastewater viral RNA levels, degree of polynomial for lag dimension, maximum lag, and adjusted pseudo R^2^. V12 is a collection of the 9 starred WWTPs with data collection ending on March 15, 2022.

WWTP code	Model type	Degree of polynomial for WW	Degree of polynomial for lag	Maximum lag (days)	Adjusted pseudo R^2^
1*	Poisson additive model	5	5	7	92.6
2*	Poisson additive model	5	5	7	94.4
3	Poisson additive model	5	4	7	90.8
4*	Poisson additive model	5	5	7	88.8
5*	Poisson additive model	5	5	7	75.4
6*	Poisson additive model	5	5	7	83.1
7*	Poisson additive model	5	3	7	65.1
8*	Poisson additive model	5	4	7	80.7
9*	Poisson additive model	5	5	7	70.4
10	Poisson regression	5	3	4	N/A
11*	Poisson regression	5	5	7	N/A
V12 (Nine sites with *)	Poisson additive model	5	5	7	95.1

**Fig 3:**
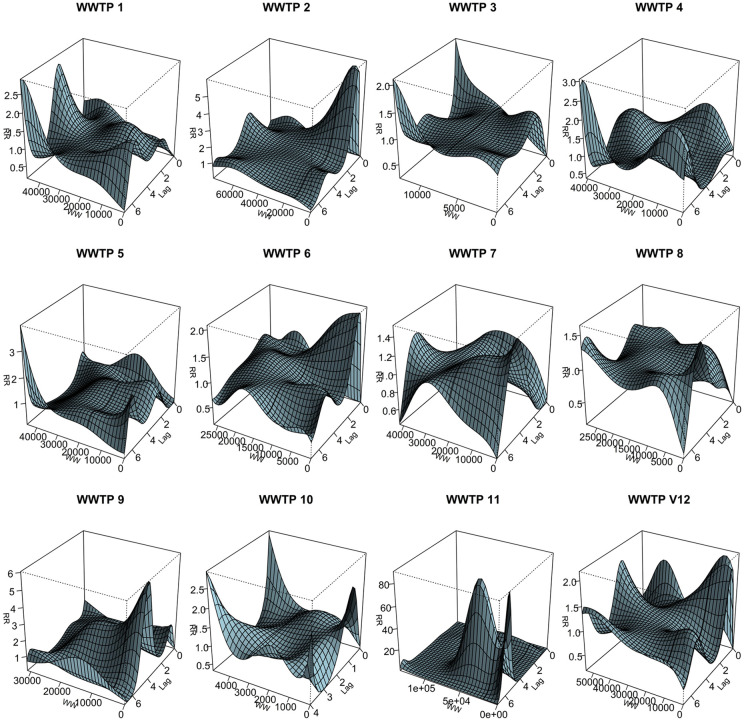
3D plots of relative risk (RR) along lags and viral RNA level (copies/100 ml; WW) for the best fitting DLNMs at each WWTP. RRs are calculated at WWTP-specific reference viral levels.

**Fig 4:**
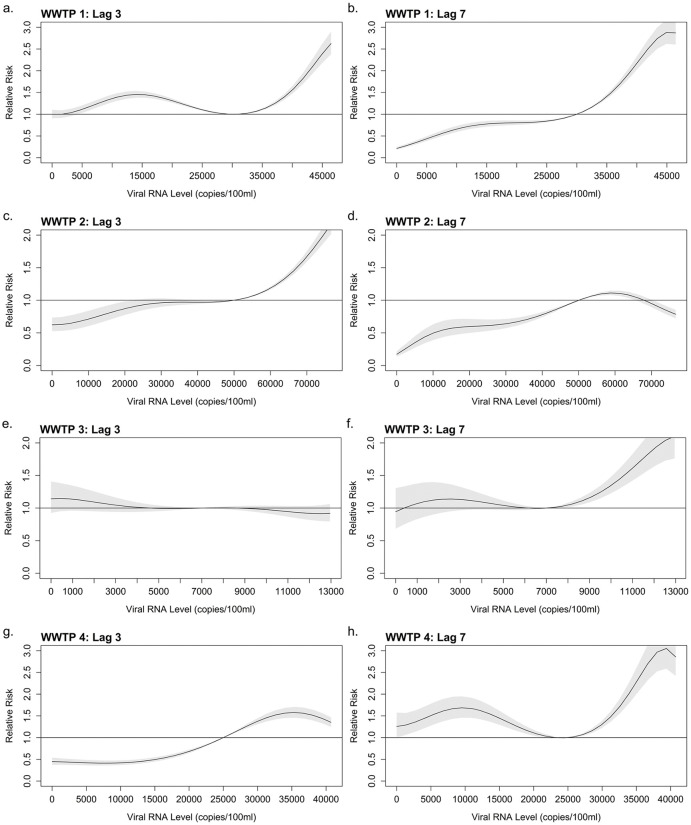
Relative risks (RRs) and associated 95% confidence intervals (shaded) for DLNMs for WWTPs 1 to 4 by wastewater viral RNA level for lags 3 and 7. RRs are calculated at WWTP-specific reference viral RNA levels.

**Fig 5:**
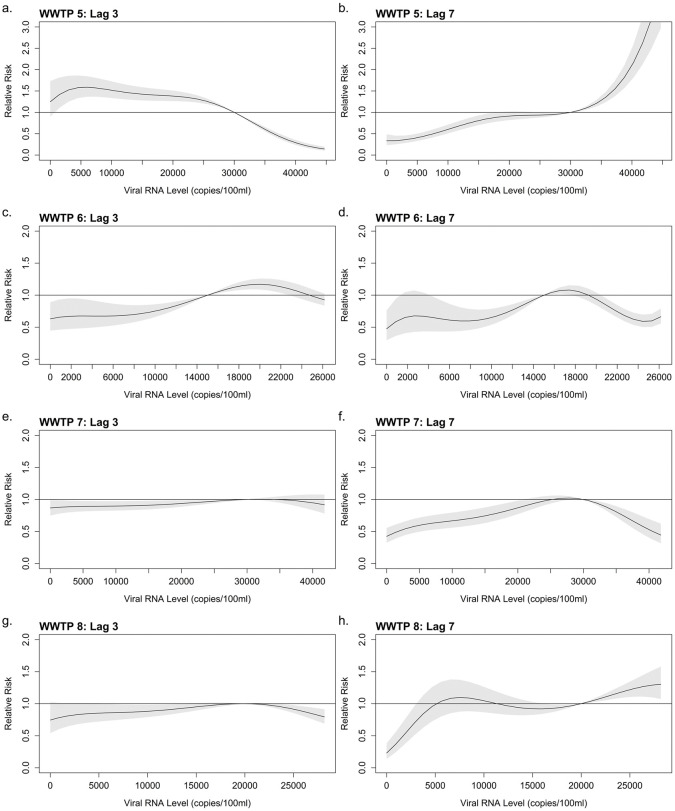
Relative risks (RRs) and associated 95% confidence intervals (shaded) for DLNMs for WWTPs 5 to 8 by wastewater viral RNA level for lags 3 and 7. RRs are calculated at WWTP-specific reference viral RNA levels.

**Fig 6:**
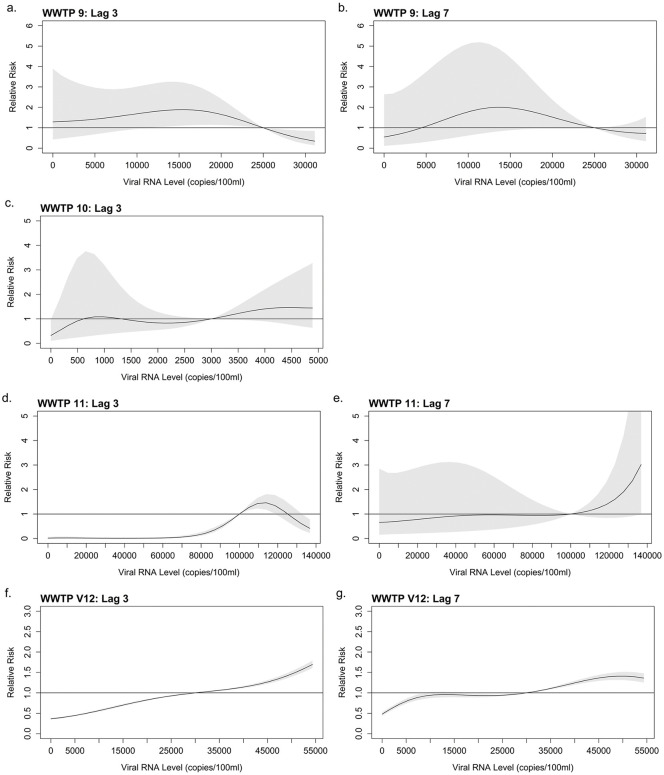
Relative risks (RRs) and associated 95% confidence intervals (shaded) for DLNMs for WWTPs 9 to V12 by wastewater viral RNA level for lags 3 and 7. RRs are calculated at WWTP-specific reference viral RNA levels.

The overall effect of wastewater viral RNA levels and clinical cases are provided as 3D plots for each WWTP (Fig 3) where RRs are provided along wastewater viral RNA level and lag. The RRs are calculated at WWTP-specific reference wastewater viral RNA levels. As these plots cannot show confidence intervals (CIs), Figs 4–6 show the specific assessments of the relationships along with 95% CIs for “slices” at lags 3 and 7 for each WWTP (except for WWTP 10 where the maximum lag was 4). The RRs are not directly comparable among WWTPs if the references are not the same. The reference is based on a central value that is WWTP-specific; however, note that the RRs from different lags from the same WWTP are comparable as they are calculated at the same reference. For example, the reference for the RR calculations for WWTP 1 and WWTP 5 are both at 30,000 copies/100 ml (point at which RR = 1 in Fig 4a and Fig 5a) whereas the reference for the RR calculation for WWTP 8 is 20,000 copies/100 ml (Fig 5g). Hence, the RRs for WWTP 1 and 5 are directly comparable to each other but not to WWTP 8.

The viral RNA levels and documented cases relationships changed across lags and WWTPs. For example, we can see for WWTP 1 (Fig 4a and 4b) that the higher levels in wastewater had a more delayed effect on increased cases (lag 7 days) but there was also a shorter delay (lag 3 days) where there was an increased risk of cases for lower viral RNA levels (e.g., around 15,000 copies/100 ml). Conversely, WWTP 2 showed that 3 days after larger viral RNA levels, the RR for cases increased. At 7 days, there was not much of an increased risk. For WWTP 5 (Fig 5a-5b), there was an initial increase in RR for lag 4 for the lower viral RNA levels but a greater increase in RR for lag 7 for the higher viral RNA levels, suggesting opposite patterns. WWTPs 9, 10, and 11 (Fig 6a-6f) have large 95% CIs, making the patterns harder to interpret. These WWTPs serve the smallest populations and there was considerable variability in the viral RNA levels. In particular, these WWTPs had some large numbers of cases in the early part of the study that did not reflect increases in wastewater viral RNA levels.

**Fig 7:**
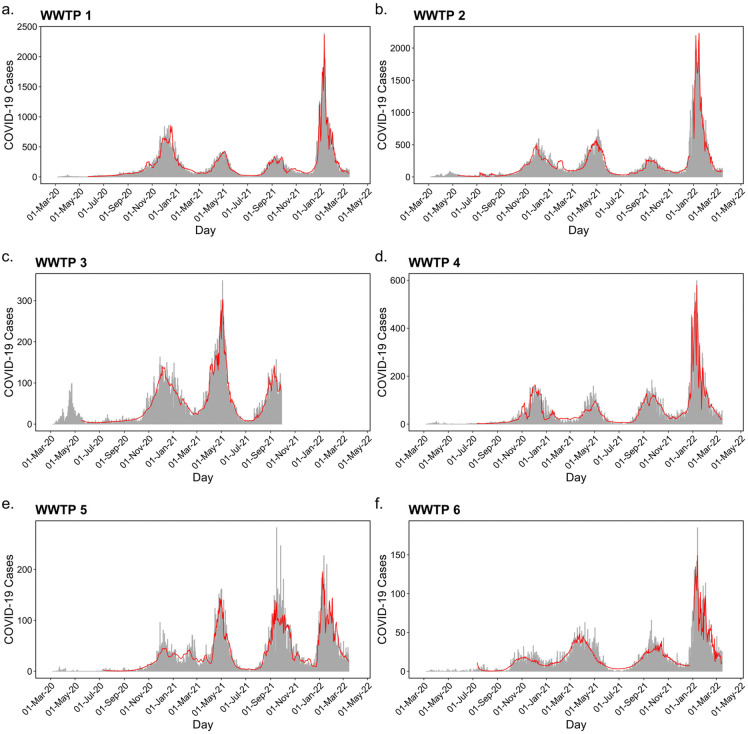
Clinical cases (grey bars) and predicted cases from models (red line) for WWTPs 1 to 6.

**Fig 8:**
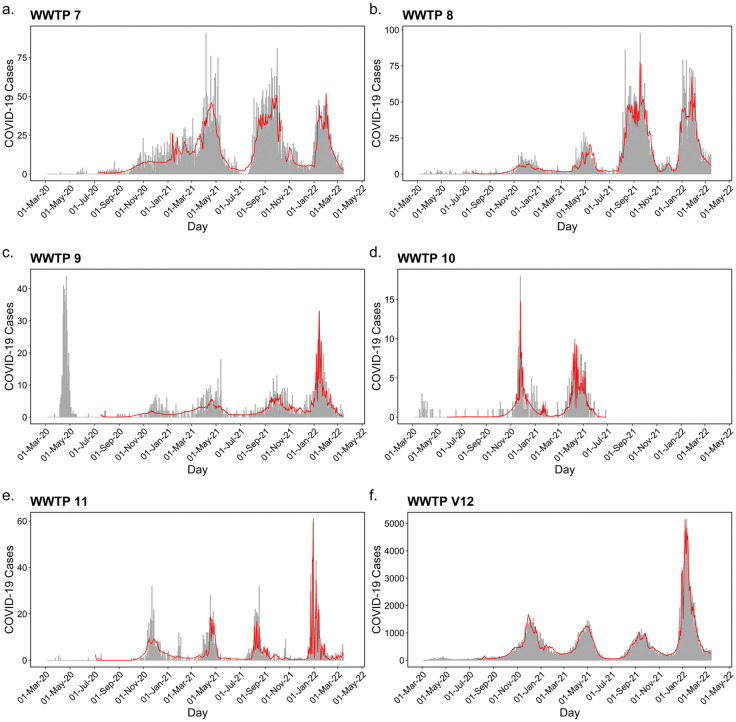
Clinical cases (grey bars) and predicted cases from models (red line) for WWTPs 7 to V12.

Figs 7 and 8 show how the predicted number of COVID-19 cases from the DLNMs would compare to the actual number cases for each WWTP. Most of the predictions followed the same general shape of the actual COVID-19 cases. In early 2021, the models for WWTPs 9, 10, and 11 tended to under-predict the actual COVID-19 cases.

## Discussion

This study examined the use of DLNMs to model the relationship between wastewater SARS-CoV-2 RNA levels and COVID-19 diagnosed cases collected as part of evolving surveillance approaches. The DLNM approach was chosen because the relationship was expected to be vary over time and to be nonlinear. Analyses were performed for each of 11 WWTPs and one virtual WWTP (representing a combination of nine tested areas) with a long study period in Alberta. While DLNMs have been proposed for wastewater viral RNA level and COVID-19 hospitalizations in a single city (Ottawa, Ontario) [13] this is the first study to examine every major urban centre in a province, representing >75% of the total province’s population, and COVID-19 clinical cases in Canada.

For most of the WWTPs, Poisson generalized additive models with a relatively high number of polynomials for the exposure-response relationship and lags were the best model. Even though the general forms of the models were the same for the WWTPs, the estimates and the resulting RRs were different for different WWTPs. It is clear from our results that not one model will be appropriate for each WWTP. Predicted values captured the trends of the actual values over time. In particular, the predictions tended to be closer to the actual values for 2021 onward which may coincide with the January 2021 change to using samples stored at 4°C to avoid the freeze-thaw cycle of frozen samples.

Generally, the adjusted pseudo-R^2^ were quite high; however, models for smaller population areas that had smaller numbers of cases did not fit as well. For example, WWTPs 11 and 10 are the resort town of Banff and a nearby municipality of Canmore, respectively. Banff is a touristy mountain area that has a large number of visitors that can number four to five times the actual population in different seasons. The population number may reflect the resident population but the wastewater viral RNA levels capture visitors as well. We also tried other models that did not involve splines of time and they did not fit well. Furthermore, there were times when the wastewater viral RNA levels did not match well with the actual cases, particularly in the beginning of the study, and that mis-match makes the modeling more challenging. Overall, for the later time period and for non-touristy areas the models fit well.

While DLNMs have been used in public health research to examine the relationships between exposures (e.g., meteorological, pollutants) and health [25–28], there is a paucity of literature that use DLNMs [13,29], or even DLMs [12,13,30–33], with wastewater. Peng et al. [13] used DLNMs to examine wastewater viral levels and COVID-19 hospitalizations in Ottawa, Canada. Our study used a similar approach to specifying the cross-basis functions as polynomials, but their single site model focused on hospitalizations and vaccination information. Specifically, their model included simulated vaccine effectiveness based on publicly available daily administered vaccine doses by public health unit and age group. These differences limit the comparability of findings from our multi-site data on COVID-19 cases. Zhang et al. [29] examined COVID-19 cases in a southern city of China and used DLNMs with data from four surveillance systems (hospital, wastewater, meteorological, internet search engine). They too used cross-basis functions as polynomials and concluded that wastewater surveillance provided a valuable early signal. Their use of DLNMs with additional predictors than wastewater viral levels limits direct comparability of model estimates.

The use of WBS for emerging pathogens of public health importance or emerging pathogens is evolving. There are many aspects to consider when building a WBS program to provide actionable information for public health officials.[34] Practical implementation, including the possibility of site-specific models, will depend on the availability of resources and expertise within a jurisdiction.

Our study has several limitations. We do not have other data to use to better inform the models like the vaccination rates in the communities over time. COVID-19 testing was generally limited to patients with infectious symptoms and required care at hospitals for COVID-19 or other medical reasons in early 2022, so while WBS still has the ability to monitor COVID-19 burden, comparing WBS with the number of new cases in current times is not possible. Our modelling does not specifically account for changes in circulating variants including Omicron. The first positive test for Omicron occurred in late November 2021. Different variants in different geographic locations could have influenced the relationship between WW and cases. Our modeling also does not specifically account for the change from frozen samples, although earlier data would not influence later predictions much because of the nature of distributed lag models. Notwithstanding these limitations, our study covers 77% of the population of Alberta and includes multiple WW facilities and a long study period.

## Conclusions

Wastewater-based surveillance is becoming a valuable public health approach for relating viral nucleic acids concentrations to clinical outcomes, potentially providing an early warning system. With relationships between wastewater viral RNA levels for SARS-CoV-2 and COVID-19 cases expected to vary over time and to be non-linear, distributed lag non-linear models provide an important analytical tool to explore these relationships. While focused on the time period when clinical testing was comprehensive, our analysis showed that while the form of the model had similarity across WWTPs, the resulting estimates were different among various communities. Our main findings indicated that the Poisson additive models worked well for all but two small areas with high numbers of tourists. These results suggest that even in the same province, site-specific analyses are essential for implementing wastewater-based epidemiology.

## Supporting information

S1 Table Akaike Information Criterion (AIC) over multiple models for WWTP 1. Lowest AIC is bolded.
(PDF)

S2 Table Akaike Information Criterion (AIC) over multiple models for WWTP 5. Lowest AIC is bolded.
(PDF)

S3 Table Akaike Information Criterion (AIC) over multiple models for WWTP 10. Lowest AIC is bolded.
(PDF)
