# Supplementary material for: Exploring the predictability of distributed lag nonlinear models using SARS-CoV-2 wastewater-based surveillance in multiple communities in Alberta, Canada
Source: PLoS One. 2026 Jul 10;21(7):e0349030. doi: 10.1371/journal.pone.0349030 (PMC13354072; doi:10.1371/journal.pone.0349030)
Supplement: S1 Table — Lowest AIC is bolded. (PDF) [file pone.0349030.s001.pdf]

S1 Table: Akaike Information Criterion (AIC) over multiple models for WWTP 1. Lowest AIC is bolded.

Model type	Maximum lag (days)	Degree of polynomial for WW	Degree of polynomial for lag				
			1	2	3	4	5
Poisson	4	1	45478.6	45385.6	45387.2	45383.7	45383.7
		2	32212.4	31634.4	31583.2	31567.0	31567.0
		3	22926.6	22421.9	22290.7	22290.2	22290.2
		4	21614.2	21025.2	20966.2	20899.4	20899.4
		5	20667.5	20064.8	20014.1	19992.0	19992.0
	5	1	45182.5	44922.8	44919.8	44799.6	44790.7
		2	32245.7	31565.6	31461.2	31400.6	31396.0
		3	22403.9	21904.1	21831.1	21775.6	21766.7
		4	21307.9	20935.5	20713.5	20681.5	20598.0
		5	20581.2	19685.0	19586.9	19515.0	19435.0
	6	1	44963.1	44301.8	44297.4	44114.0	44114.3
		2	32377.8	31461.7	31331.8	31210.4	31212.4
		3	22048.6	21627.0	21498.7	21398.9	21396.5
		4	21146.2	20839.2	20602.1	20434.2	20397.4
		5	20463.6	19360.2	19334.9	19155.4	19085.7
	7	1	44762.9	43320.2	43301.1	43207.8	43209.8
		2	32567.5	30970.5	30935.9	30732.4	30727.6
		3	21841.4	21280.3	21132.1	20889.5	20829.0
		4	21090.4	20590.3	20410.3	19903.2	19830.4
		5	20282.5	19058.2	18696.9	18239.3	18081.8
Poisson additive	4	1	41500.1	41426.4	41427.7	41425.8	41425.8
		2	27187.6	26492.2	26418.9	26397.8	26397.8
		3	20641.0	20169.1	20043.3	20042.6	20042.6
		4	19716.1	19228.0	19167.3	19110.7	19110.7
		5	19004.2	18533.8	18484.7	18452.7	18452.7
	5	1	41380.0	41163.0	41160.9	41056.4	41048.4
		2	27168.7	26319.6	26216.1	26129.9	26123.9
		3	20207.8	19745.0	19676.9	19628.3	19620.0
		4	19468.8	19184.7	18961.2	18926.1	18839.2
		5	19010.4	18296.5	18195.5	18133.5	18063.4
	6	1	41323.5	40746.6	40743.2	40574.8	40574.8
		2	27331.4	26172.5	26050.1	25885.7	25884.4
		3	19938.3	19548.5	19439.7	19345.8	19346.4
		4	19345.4	19133.8	18899.1	18750.2	18725.4
		5	19067.1	18059.6	18029.6	17872.4	17820.3
	7	1	41291.6	39928.4	39908.2	39811.6	39813.6
		2	27559.2	25627.1	25592.9	25317.9	25314.0
		3	19797.0	19284.7	19172.0	18955.8	18900.3
		4	19323.1	18977.7	18826.9	18413.6	18349.4
		5	19030.7	17882.3	17510.9	17123.5	**16946.6**
