# Supplementary material for: Exploring the predictability of distributed lag nonlinear models using SARS-CoV-2 wastewater-based surveillance in multiple communities in Alberta, Canada
Source: PLoS One. 2026 Jul 10;21(7):e0349030. doi: 10.1371/journal.pone.0349030 (PMC13354072; doi:10.1371/journal.pone.0349030)
Supplement: S2 Table — Lowest AIC is bolded. (PDF) [file pone.0349030.s002.pdf]

S2 Table: Akaike Information Criterion (AIC) over multiple models for WWTP 5. Lowest AIC is bolded.

Model type	Maximum lag (days)	Degree of polynomial for WW	Degree of polynomial for lag				
			1	2	3	4	5
Poisson	4	1	14228.4	13869.0	13870.1	13872.0	13872.0
		2	9471.6	9266.8	9269.3	9255.7	9255.7
		3	8275.2	8194.1	8193.0	8194.3	8194.3
		4	8094.6	8014.5	8019.7	8006.2	8006.2
		5	7986.9	7913.6	7919.5	7917.6	7917.6
	5	1	14071.1	13461.2	13456.3	13456.8	13457.6
		2	9387.1	9149.4	9131.8	9124.5	9115.7
		3	8237.0	8096.0	8072.7	8073.5	8065.8
		4	8054.4	7953.8	7876.5	7856.0	7848.6
		5	7937.1	7794.2	7712.3	7685.8	7682.7
	6	1	13941.3	13322	13317.8	13318.1	13312.3
		2	9334.6	9135.2	9107.3	9106.6	9089.5
		3	8220.5	8090.9	8074.3	8057.2	8045.6
		4	8039.8	7945.9	7901.6	7878.4	7809.5
		5	7916.3	7783.9	7755.9	7681.4	7613.3
	7	1	13793.0	13063.1	13062.4	13011.4	13001.5
		2	9287.0	9061.0	9044.6	9000.3	8991.5
		3	8203.7	8034.0	8024.2	8011.6	7991.9
		4	8018.8	7899.5	7863.2	7830.5	7826.6
		5	7899.1	7696.2	7657.0	7589.1	7559.6
Poisson additive	4	1	13550.6	13319.9	13321.2	13323.2	13323.2
		2	9586.9	9386.9	9389.4	9376.0	9376.0
		3	8153.8	8076.7	8073.3	8072.9	8072.9
		4	7928.2	7841.7	7846.0	7831.1	7831.1
		5	7788.3	7720.1	7724.9	7722.4	7722.4
	5	1	13409.8	12976.5	12973.3	12973.9	12975
		2	9482.0	9247.0	9229.8	9222.8	9213.9
		3	8099.0	7968.0	7941.2	7944.1	7937.8
		4	7893.6	7811.1	7729.8	7711.1	7702.5
		5	7741.9	7644.4	7562.5	7539.3	7534.0
	6	1	13292.7	12848.1	12841.3	12841	12835.7
		2	9411.2	9214.5	9189.2	9188.3	9171.3
		3	8065.3	7943.0	7926.7	7902.2	7888.9
		4	7882.2	7794.4	7749.0	7720.4	7647.1
		5	7722.4	7628.2	7605.6	7526.7	7457.4
	7	1	13161.9	12603.1	12600.1	12555.4	12545.0
		2	9344.0	9118.0	9103.6	9058.4	9048.7
		3	8033.2	7873.0	7863.2	7850.5	7827.2
		4	7861.5	7742.8	7701.0	7663.6	7656.6
		5	7710.0	7544.2	7506.2	7430.5	**7394.9**
