# Supplementary material for: Exploring the predictability of distributed lag nonlinear models using SARS-CoV-2 wastewater-based surveillance in multiple communities in Alberta, Canada
Source: PLoS One. 2026 Jul 10;21(7):e0349030. doi: 10.1371/journal.pone.0349030 (PMC13354072; doi:10.1371/journal.pone.0349030)
Supplement: S3 Table — Lowest AIC is bolded. (PDF) [file pone.0349030.s003.pdf]

S3 Table: Akaike Information Criterion (AIC) over multiple models for WWTP 10. Lowest AIC is bolded.

Model type	Maximum lag (days)	Degree of polynomial for WW	Degree of polynomial for lag				
			1	2	3	4	5
Poisson	4	1	845.8	847.5	834.7	836.7	836.7
		2	837.1	839.1	828.9	830.9	830.9
		3	833.6	836.7	822.0	826.6	826.6
		4	827.7	830.1	819.0	823.2	823.2
		5	828.6	827.7	**814.1**	820.0	820.0
	5	1	845.2	846.5	838.3	833.8	835.8
		2	836.9	838.5	832.9	830.2	832.0
		3	833.0	835.9	830.1	824.8	829.2
		4	826.4	830.6	827.0	824.3	829.3
		5	824.2	828.2	827.4	822.4	826.4
	6	1	844.7	846.0	845.9	835.0	836.0
		2	836.2	838.2	839.3	831.5	834.3
		3	832.5	835.3	837.2	826.1	828.9
		4	825.5	831.1	830.5	825.4	829.5
		5	826.5	833.5	826.3	823.4	830.2
	7	1	843.9	845.2	846.5	841.7	835.2
		2	836.0	839.3	839.5	836.9	833.1
		3	831.5	836.0	837.1	836.4	824.6
		4	823.9	830.5	833.1	832.9	822.7
		5	825.9	833.6	837.2	835.7	821.8
Poisson additive	4	1	849.7	851.4	839.2	841.1	841.1
		2	842.5	844.4	834.6	836.6	836.5
		3	840.1	843.1	828.9	833.3	833.1
		4	828.7	828.6	817.5	821.9	821.8
		5	831.8	829.8	816.6	822.5	822.4
	5	1	848.6	850.0	842.2	837.9	839.8
		2	842.0	843.7	838.4	835.9	837.6
		3	839.4	842.2	836.4	831.1	835.4
		4	826.3	828.2	824.6	821.9	826.9
		5	827.9	830.3	828.3	821.8	825.7
	6	1	847.4	848.9	848.9	838.3	839.4
		2	840.9	843.1	844.1	836.6	839.3
		3	838.4	840.9	842.5	831.3	834.1
		4	825.7	829.9	828.8	824.2	827.6
		5	827.6	832.2	822.0	819.2	825.8
	7	1	846.0	847.5	848.8	844.1	838.0
		2	840.2	843.7	843.5	841.1	837.6
		3	837.1	841.3	842.1	841.5	829.7
		4	823.8	829.4	831.1	829.7	818.8
		5	826.0	831.9	833.5	831.3	818.3
